# Short-term weight-centric effects of tea or tea extract in patients with metabolic syndrome: a meta-analysis of randomized controlled trials

**DOI:** 10.1038/nutd.2015.10

**Published:** 2015-06-15

**Authors:** X Zhong, T Zhang, Y Liu, X Wei, X Zhang, Y Qin, Z Jin, Q Chen, X Ma, R Wang, J He

**Affiliations:** 1 Department of Health Statistics, Second Military Medical University, Shanghai, China; 2 School of Medicine, Shanghai Jiaotong University, Shanghai, China

## Abstract

To evaluate the weight-centric effect of tea or tea extract in participants with metabolic syndrome (MetS), we performed electronic searches in PubMed, EmBase and the Cochrane Library to identify eligible randomized controlled trials (RCTs) comparing tea or tea extract vs a control group. A direct meta-analysis using random-effects model was conducted to pool the standardized mean difference regarding body mass index (BMI), body weight and waist circumference. Study quality was assessed by using the Jadad scale. Pre-specified subgroup and sensitivity analyses were conducted to explore potential heterogeneity. A total of five RCTs involving 338 adult individuals were included. Given the high heterogeneity observed in the overall pooled analysis, we separated the included subjects into two subgroups. Ingestion of tea or tea extract significantly reduced BMI (subgroup 1: −1.60, 95% confidence interval (CI), −2.05 to −1.14; subgroup 2: −0.40, 95% CI, −0.69 to −0.12) and body weight (subgroup 1: −4.14, 95% CI, −4.85 to −3.43; subgroup 2: −0.35, 95% CI, −0.68 to −0.02). This meta-analysis suggests that tea or tea extract has favorable weight-centric effects in MetS patients. Additional large RCTs specifically designed to evaluate the effect on anthropometric measurements are needed to further confirm these findings.

## Introduction

Metabolic syndrome (MetS) is usually diagnosed by a co-occurrence of three out of five of the following medical conditions: abdominal obesity, elevated glucose, elevated blood pressures, high triglycerides and low high-density lipoprotein-cholesterol levels.^1, 2^ The International Diabetes Federation estimates that one-quarter of the world's adult population has MetS.^3^ In the last decade, MetS has been increasing to the point of being regarded as an epidemic.^1^ Abdominal obesity, one of its common components, is associated with a substantially increased risk for type 2 diabetes, in fact 80% of patients with type 2 diabetes are overweight or obese. Recently Scheen and Van Gaal^4^ even proposed a shift from a glucocentric to a weight-centric management for patients with type 2 diabetes. Moreover, with a modest weight loss, both hypertensive individuals and subjects at risk of developing hypertension can observe a significant reduction in the blood pressure.^5^ Meanwhile, a weight loss of as small as 5–10% of body weight can significantly reduce triglycerides and increase high-density lipoprotein-cholesterol.^6^ Therefore, this weight loss appears specific to the weight but results in an improvement of many obesity-related conditions, including various abnormal components of the MetS and development of diabetes.^7^

Tea, mostly in the form of green (20% of produced tea) or black tea (78%), is the most widely consumed beverage in the world, second only to water.^8, 9, 10^ Tea contains many compounds, especially polyphenols, known as catechins. Green and black teas are similar in total polyphenol content.^9^ In fact, green tea has many kinds of catechins, such as epigallocatechin gallate, epicatechin-3-gallate, epigallocatechin and epicatechin, whereas black tea contains more of theaflavins and thearubigins, which are due to extra oxidation and partial polymerization of catechins.^9, 10^ Catechins like epigallocatechin gallate may be useful for preventing and treating obesity by virtue of a potential mechanism that involves inhibition of adipocyte differentiation and proliferation,^11^ reduced fat absorption,^12^ inhibition of catechol-o-methyl-transferase,^11^ increased fat oxidation,^9, 10^ increased energy expenditure,^13^ and increased utilization of fat.^12^ Given its huge consumption as the cheapest human beverage, any small effects of tea at a population level would lead to a huge impact on public health, even if we focus on obesity management. Recently human research on tea's multifaceted beneficial effects has gained more interest for a diversity of clinical disorders such as cancer, cardiovascular diseases and diabetes.^9, 10^ Among them, a growing body of research mentions positive effects of green tea catechins on metabolic parameters. A previous meta-analysis of 17 randomized controlled trials (RCTs)^14^ suggested that green tea decreased fasting glucose and glycated hemoglobin concentrations by −0.09 mmol l^−1^ (95% CI: −0.15, −0.03 mmol l^−1^; *P*<0.01) and −0.30% (95% CI: −0.37, −0.22% *P*<0.01) in healthy people and those at risk of MetS, respectively. However, this analysis neither included MetS patients nor reported anthropometric measurements. In similar subjects without MetS (healthy or overweight/obese people), another three meta-analyses based on RCTs^15, 16, 17^ showed that green tea had exerted inconsistent effects on body weight loss and weight maintenance, which might relate to ethnicity and co-intervention. In recent years several modest RCTs have been conducted in MetS patients to investigate the effects of tea or tea extract on anthropometric measurements. Therefore, given these data, we aimed to carry out the first meta-analysis to quantitatively assess the effects of tea or tea extract in MetS patients based on these published RCTs.

## Materials and methods

### Search strategy and selection criteria

RCTs to investigate the comparative effects of tea or tea extract were eligible for inclusion in our analysis, without any restriction on language or publication status. We electronically searched the PubMed (from 1965 to November 2014), EmBase (from 1965 to November 2014) and Cochrane Library databases using the search terms ‘Metabolic Syndrome' AND Randomized Controlled Trial AND (‘Tea' or ‘tea extract' or ‘catechin' or ‘epigallocatechin gallate' or ‘Camellia sinensis' or ‘tea polyphenol'). We also further conducted manual searches of reference lists from relevant original and review articles.

Eligible RCTs had to meet the following inclusion criteria: (i) had to be published as an original article; (ii) study participants had to be adults with MetS; (iii) should have evaluated the use of tea or tea extract as one of study interventions; and (iv) should have reported data on at least one of the following end points: (1) body mass index (BMI), (2) body weight, (3) waist circumference (WC) or (4) waist-hip-ratio. Both parallel and crossover trials were eligible for inclusion, but ultimately no eligible crossover trials were identified. If more than one article reported data from the same study, the most recent and complete articles were included.

### Data extraction and risk of bias assessment

The following information was extracted from each eligible study: (i) first author's surname, (ii) publication year; (iii) sample size; (iv) study treatment duration; (v) baseline characteristics (gender, age, co-morbidities, MetS diagnostic criteria and *et al.*); (vi) type of diet, lifestyle and exercise status; (vii) study treatment description, and (viii) parameters of end points (BMI, body weight, WC and waist-hip-ratio) and adverse event data.

Data were extracted using a standardized data-recording form and risk for bias was assessed according to the Preferred Reporting Items for Systematic Reviews and Meta-Analyses guidelines.^18^ The study search and selection, data extraction and risk of bias assessment were conducted independently by two investigators (XZ and XZ). Information was checked and adjudicated independently by an additional investigator (JH) until agreement was achieved.

### Statistical analysis

In order to report standardized mean difference between two treatments for each study, we extracted number of subjects, arithmetic means and s.d. or s.e. from the included RCTs. When only baseline and follow-up parameter levels were obtained, the s.d. with regard to changes in each parameter since baseline were derived with imputed correlation coefficients according to the Cochrane Handbook.^19^

Both fixed-effect and random-effects models were used to evaluate the overall pooled standardized mean difference. Given present heterogeneity, however, the results of random-effects models were used to draw any study conclusions. Heterogeneity between the studies was evaluated by the *Q*-test and *I*-squared (*I*^2^) statistic.^20, 21^ These indices assess the percentage of variability across studies, which is attributable to heterogeneity rather than chance. Statistical heterogeneity was considered significant when *P*<0.10 for the *Q*-test or *I*^2^>50%.

Funnel plots for BMI, weight and WC were drawn and the Egger's regression test^22^ was used to statistically assess publication bias. The following subgroup analyses were performed according to stratification factors such as concomitant non-pharmacologic or pharmacologic intervention (yes vs no), type of tea (Puerh tea vs green tea), study country (Asian vs Non-Asian), MetS diagnostic criteria (International Diabetes Federation vs Other), study treatment duration (2–3 vs 6 months) and Jadad score (<4 vs ⩾4; Jadad scale of 0–5 points^23^) to explore possible heterogeneity sources. Owing to obvious heterogeneity observed in the study, we further conducted subgroup meta-analyses to find potential heterogeneity sources (co-intervention, namely concomitant management plan plus five MetS risk factors (yes vs no)). In each specific subpopulation, the effects of tea or tea extract over control were evaluated. Statistical analyses were performed using R 3.0.3 with package ‘meta'.^24^ Besides, sensitivity analysis was performed after removing any trials in which extra concurrent non-pharmacologic intervention was added. The R codes are available from the authors upon request.

## Results

### Search results and characteristics of the studies

**Figure 1:**
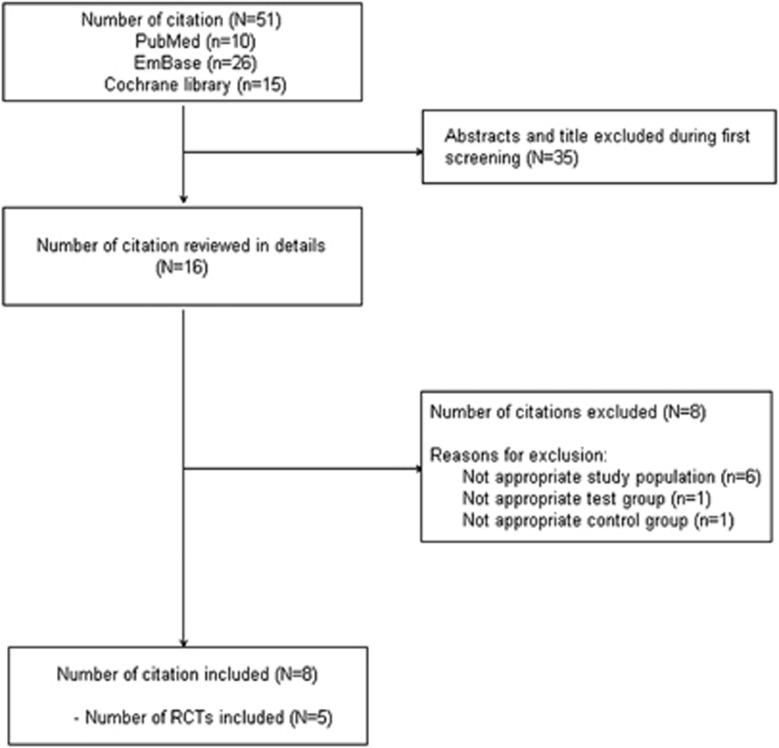
Flow diagram of the studies search and selection process.

A total of 51 reports were initially identified according to our search strategy. We excluded 35 reports after title and abstract screening and then conducted detailed review for the remaining 16 reports. Finally five distinct RCTs (338 individuals) with eight reports^25, 26, 27, 28, 29, 30, 31, 32^ met our inclusion criteria and were included in our meta-analysis. Detailed processes of the relevant study selection are shown in Figure 1.

Table 1 presented study and participant characteristics for the included five trials. Two out of five trials were conducted in the Asian population^25, 32^ and the other three in non-Asian subjects.^26, 27, 28, 29, 30, 31^ Three trials used International Diabetes Federation's MetS diagnosis criteria^30, 31, 32^ and another two used Chinese criteria^25^ or Adult Treatment Panel III.^26, 27, 28, 29^ Study treatments in four trials lasted for 2 or 3 months except one RCT^31^ running for 6 months. Mean ages were all more than 40 years and both sexes were present in all trials. In the trial of Belcaro *et al.*^31^ it was seen that subjects had a pre-defined pharmacologic intervention plan: changes in diet, thus avoiding junk food and limiting high-calorie elements, and a precise individual exercise plan. Hence this RCT was excluded from the sensitivity analysis. For two trials^25, 26, 27, 28, 29, 30, 31^ whose part of measured data for this meta-analysis were not provided in the published articles, we contacted the articles' correspondence author via E-mail with a request, but have not received any replies so far. For a study,^26, 27, 28, 29^ the tea and tea extract group in a study are pooled into one tea group and the pooled mean and s.d. are used.

Table 2 presents the risk assessment for bias in the five trials according to the Preferred Reporting Items for Systematic Reviews and Meta-Analyses guidelines. Four included RCTs reported specific randomization methods or details,^25, 26, 27, 28, 29, 30, 32^ and no trials stopped early. There are two RCTs^25, 32^ using double-blinded design with a Jadad score of five points, two^26, 27, 28, 29, 31^ with single-blinded (three points) design and one^30^ with open-labeled design (two points). None of the RCTs clearly specified whether data collectors and outcome assessors were blinded to study data. In general, the overall quality of included RCTs could be rated as good.

### Reduction in BMI

**Figure 2:**
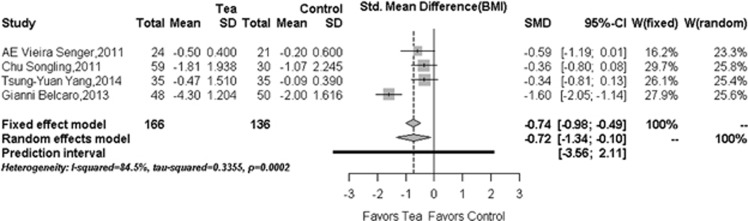
Forest plots in the meta-analysis of effect of tea or tea extract on BMI changes. Sizes of the data markers indicate the weight of each study in this analysis. The diamond represents the overall estimated effects in each model. SMD, standardized mean difference.

Four^25, 30, 31, 32^ out of five included RCTs reported BMI data (*N*=302) and each study showed varied reduction of BMI levels after treatment of tea or tea extract. As shown in Figure 2, our overall pooled analysis indicated that tea or tea extract significantly lowered BMI level (−0.72, 95% CI: −1.34 to −0.10; *P*=0.0219) when compared with the control group. However, a significant heterogeneity in overall pooled analysis (*I*^2^=84.5%, Cochran's test with *P*=0.0002) was observed. We then used the specified subgroup analysis results (subgroup 1: with co-intervention in subjects with five MetS risk factors; subgroup 2: no co-intervention in subjects with less MetS risk factors) to report data. Ingestion of tea or tea extract significantly reduced BMI in subgroup 1 (−1.60, 95% CI, −2.05 to −1.14) and subgroup 2 (−0.40, 95% CI, −0.69 to −0.12) as well. This conclusion holds true in the sensitivity analysis (−0.40, 95% CI: −0.69 to −0.12) and almost all other subgroups (see Table 3 for details). When the study of Belcaro *et al.*^31^ was excluded in the sensitivity analysis, the heterogeneity test yielded a value of *I*^2^=0% and *P*=0.7932 in Cochran's test as well. The subgroup analyses equally showed the influence by the same study (Table 3).

### Reduction in body weight

**Figure 3:**
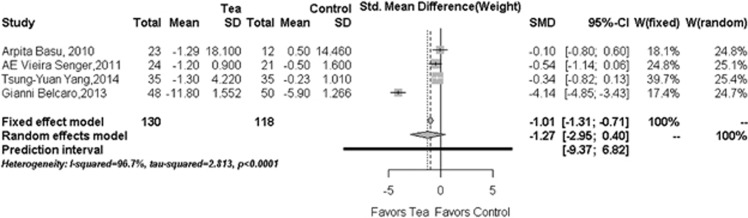
Forest plots in the meta-analysis of effect of tea or tea extract on body weight changes. Sizes of the data markers indicate the weight of each study in this analysis. The diamond represents the overall estimated effects in each model. SMD, standardized mean difference.

There are four included RCTs (*N*=248)^26, 27, 28, 29, 30, 31, 32^ reporting body weight change data. They all consistently showed the reduction of body weight levels after treatment with tea or tea extract. Our overall pooled analysis of the random-effects model did not indicate that tea or tea extract could significantly lower body weight level (−1.27, 95% CI: −2.95 to 0.40; *P*=0.1358) when compared with the control group (Figure 3); however, after considering the source of heterogeneity, a statistically significant net change was found in both subgroups (subgroup 1:−4.14, 95% CI, −4.85 to −3.43; subgroup 2: −0.35, 95% CI, −0.68 to −0.02) (see Table 3 for details) and in the sensitivity analysis (−0.35, 95% CI: −0.68 to −0.02).

### Change in WC

**Figure 4:**
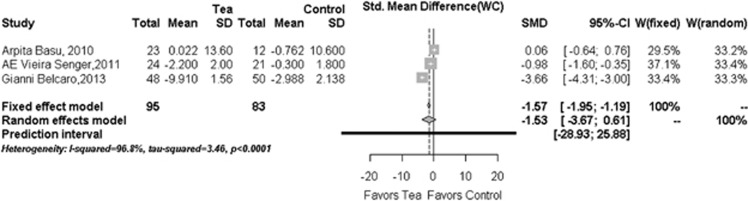
Forest plots in the meta-analysis of effect of tea or tea extract on WC changes. Sizes of the data markers indicate the weight of each study in this analysis. The diamond represents the overall estimated effects in each model. SMD, standardized mean difference.

Only three studies (*N*=178)^26, 27, 28, 29, 30, 31^ provided WC change data. No statistically significant net difference between the tea or tea extract group and control group was found in overall pooled analysis (−1.53, 95% CI: −3.67 to 0.61; *P*=0.1618) (Figure 4) and in some subgroups stratified by co-intervention plan or treatment duration (data not shown).

### Adverse event

Only one adverse event (diarrhea) was reported from the Pu'er tea extract group in the study of Chu *et al.*^25^ and the subject dropped out from study owing to this event.

### Publication bias

Funnel plots and Egger's tests found no significant publication bias in the current meta-analysis of BMI, body weight and WC (Egger's test: *P*=0.8824, 0.4589 and 0.8215, respectively).

## Discussion

MetS is a multifaceted health problem. A healthy lifestyle and lifestyle modification including weight reduction is likely the most effective in controlling MetS.^1, 2, 33^ However, it is difficult to initiate and maintain healthy lifestyles, in particular with the recidivism of obesity in most patients who lose weight. MetS cannot be treated with a single pharmacological agent.^1, 2, 34^ Pharmacological agents that deal with obesity, diabetes, hypertension and dyslipidemia are usually administered in combination among MetS patients. However, their safety concerns are often observed and reported in various studies.^1, 2, 4^ Drinking beverage tea has been considered a health-promoting habit since ancient times. Notably in the last 30 years, there is more and more scientific evidence available to support its health benefits in a diversity of major chronic diseases, including multifaceted positive effects on metabolic conditions, for example, improving blood glucose and insulin sensitivity^14^ and reducing body weight, BMI^16, 17^ and so on. Hence we performed this meta-analysis to quantify its weight-centric effects in MetS patients.

To our knowledge, this is the first meta-analysis to investigate the effect of tea or tea extract on anthropometric measurements (BMI, body weight and WC) in MetS patients. Our results suggested that consumption of tea or tea extract had favorable weight-centric effects, for example, significantly reducing BMI and body weight. Meanwhile, various subgroup analyses and sensitivity analysis showed consistent trend of effects with supportive data. Uncommon gastrointestinal side effects (for example, diarrhea) with tea ingestion were reported in the included RCTs, which was in line with those observed in another meta-analysis conducted among overweight or obese adults (1562 patients in 14 included RCTs).^15^

It was noted that the current analyses showed slight inconsistencies in the effects of tea or tea extract on anthropometric measures in the subgroup analyses. The ingestion of tea or tea extract significantly decreased BMI and body weight in any subgroups as long as the study of Belcaro *et al.*^31^ was excluded. On the other hand, in any subgroups that contained this study,^31^ a high heterogeneity and a possible nonsignificant reduction were universally observed. This study was therefore investigated and showed differences. First, all of its study patients reached the borderline for all of five MetS risk factors. This suggests that the beneficial effect of tea or tea extract on BMI and body weight might be more pronounced in subjects with more MetS risk factors. An equal finding was reported in a recent meta-analysis of green tea's effect on fasting glucose in subjects at risk of MetS: the subjects with MetS risks reported more reduction of fasting glucose than healthy subjects.^14^ Conversely, in another meta-analysis among overweight or obese adults, only a small, statistically nonsignificant and likely clinically unimportant weight loss (−0.04 kg, 95% CI: −0.50 to 0.40; *P*=0.88) was found in a subject subgroup outside of Japan.^15^ A randomized placebo-controlled trial in overweight or obese postmenopausal women that evaluated epigallocatechin gallate alone showed nonsignificant decreases in BMI and body weight when compared with placebo.^33^ Second, the study of Belcaro *et al.*^31^ has a longer tea drinking duration of 6 months. However, as expected from the change in fasting glucose and insulin levels,^14^ the longer tea-drinking duration would not give rise to more effects on BMI and body weight. Third, a concomitant management plan was in particular noted in this study.^31^ There is no doubt that calorie restriction (for example, 500 kcal per day deficit) and increased physical activity could play a positive role,^35^ which might reduce more the body weight in both groups. In a previous meta-analysis report,^16^ green tea catechins with caffeine significantly decreased BMI and body weight than caffeine alone. This suggested that the effect of green tea catechins might be due to the combination, rather than due to any single catechin.^16^ In fact, green tea and black tea have many kinds of catechins and a small amount of natural caffeine as well.^9, 10^ More varieties of catechins and other compounds and also combination with a pre-defined management plan may further increase the existing weight loss. Therefore, high heterogeneity due to concomitant intervention echoed the necessity of our pre-defined subgroup analyses. Taken together, this study was an important potential source of heterogeneity. Hence, besides the subgroup analysis based on co-intervention plus five MetS risk factors (yes vs no), a sensitivity analysis was done with this study removed and contributed consistent results with the overall pooled analysis.

Our study has several potential limitations that could affect the meta-analysis results. First, our study was based on reported aggregate data from very few included studies rather than individual patient data, which may not provide robust estimation for the comparative efficacy. The quality of our study was determined by the quality of the individual studies included. Therefore, we only included RCTs in our analysis. RCTs might provide a possibility to estimate the net effect of treatment with tea and tea extract when all controls except uniform background therapies are non-active. Second, these five RCTs were conducted with either possible concomitant intervention or not, different patient characteristics and MetS risk factors, MetS diagnosis criteria, treatment duration, ethnicity/regions, dosage regimen and so on, all of which could be major sources of heterogeneity in our analysis. Therefore, the random-effects model was used as the primary analysis in our study and multiple subgroup analyses were considered. Even so, the study findings should be applied with caution. Third, data availability is limited. Not all included RCTs have four anthropometric measurements of interest reported. Waist-hip-ratio was not pooled, as only one study^25^ provided these data. Because of the small number of included studies and lack of a uniform dosing unit, we could not assess a dose–response relation through meta-regression. Finally, the follow-up duration of all of the included RCTs was within 6 months. One cross-sectional study by Wu *et al.*^36^ reported that adults who routinely (that is, ⩾1 time per week) consumed green, oolong or black tea for >10 years have lower body fat percentages and smaller WCs than non-habitual tea drinkers, which suggested that tea consumption may play a positive role in long-term weight loss and weight maintenance. However, further prospective research is warranted in the future. Moreover, long-term clinical event outcomes rather than surrogate end points and cost-effectiveness end points would be critically important for a more comprehensive evaluation.

In summary, our study reported that tea or tea extract results in a significant decrease in BMI and body weight as well. Additional large-scale RCTs designed to evaluate the weight-centric effects are needed to confirm these findings within specific clinical settings and subgroups.

## Figures and Tables

**Table 1:** Characteristic of RCTs included in the meta-analysis

*First author*^*ref*^	*Sample size*	*Treatments duration (months)*	*Population*	*Tea or tea extract group*	*Control group*	*Type of diet, lifestyle and exercise status*
Chu *et al.*^25^	90	3	Chinese, overweight/obese and hyperlipidemia, MetS according to Chinese diagnosis criteria; 50 years of mean age (18 years)	1000 mg Puerh tea water extract filled in eight capsules	Placebo (cellulose capsule)	Adviced on a healthy diet
Basu *et al.*^26, 27, 28, 29^	35	2	Overweight/obese, MetS according to ATP III; matched for age (±5 years) and gender; 42.5 years of mean age (21 years)	Green tea (4 cups, 928 mg catechins) or green tea extract (2 capsules, 870 mg catechins)	Water (4 cups)	Maintained usual diet, physical activity and lifestyle
Vieira Senger *et al.*^30^	45	2	MetS according to IDF diagnosis criteria; elderly people (60 years)	Three sachets of 1000 mg of green tea	No treatment	Maintained usual diet, physical activity and lifestyle
Belcaro *et al.*^31^	98	6	Borderline profile for all five MetS factors according to IDF diagnosis criteria; 46.5 years of mean age (45 years)	300 mg green tea extract devoid of caffeine and formulated in lecithin	Placebo (blank formulation)	Management plan (made changes in diet, thus avoiding junk food and limiting high-calorie elements and also a precise individual exercise plan)
Yang *et al.*^32^	70	3	Chinese, overweight/obese, MetS according to IDF diagnosis criteria; 59.2 years of mean age	999 mg Puerh tea extract in three capsules	Placebo (dextrin)	No change for dietary intervention or previous medication(s)

Abbreviations: ATP III, Adult Treatment Panel III; IDF, International Diabetes Federation; MetS, metabolic syndrome; RCTs, randomized controlled trials.

**Table 2:** Risk for bias assessment in selected randomized controlled trials

*First author*^*ref*^	*Concealment of randomization*	*Stopped early*	*Participants blinded*	*Health care providers blinded*	*Data collectors blinded*	*Outcome assessors blinded*
Chu *et al.*^25^	Yes	No	Yes	Yes	Not informed	Not informed
Basu *et al.*^26, 27, 28, 29^	Yes	No	Yes	No	Not informed	Not informed
Vieira Senger *et al.*^30^	Yes	No	No	No	Not informed	Not informed
Belcaro *et al.*^31^	Not informed	No	Yes	No	Not informed	Not informed
Yang *et al.*^32^	Yes	No	Yes	Yes	Not informed	Not informed

**Table 3:** Subgroup analyses of BMI and body weight stratified by pre-defined study characteristics

	*BMI*				*Body weight*			
*Study characteristics*	*Number of subjects*	*Net change (95% CI)*	*Test of heterogeneity*		*Number of subjects*	*Net change (95% CI)*	*Test of heterogeneity*	
			I^2^ (%)	P*-value*			I^2^ (%)	P*-value*
								
*Co-intervention plus five MetS risk factors*								
Yes	98	−1.60 (−2.05, −1.14)	NA	1.0000	98	−4.14 (−4.85, −3.43)	NA	1.0000
No	204	−0.40 (−0.69, −0.12)	0	0.7932	150	−0.35 (−0.68, −0.02)	0	0.6478
								
*Concomitant management plan*								
Yes	98	−1.60 (−2.05, −1.14)	NA	1.0000	98	−4.14 (−4.85, −3.43)	NA	1.0000
No	204	−0.40 (−0.69, −0.12)	0	0.7932	150	−0.35 (−0.68, −0.02)	0	0.6478
								
*Type of tea or tea extract*								
Puerh tea	159	−0.35 (−0.67, −0.03)	0	0.9568	70	−0.34 (−0.82, 0.13)	NA	1.0000
Green tea	143	−1.11 (−2.10, −0.12)	85.5	0.0086	178	−1.59 (−4.00, 0.82)	97.4	<0.0001
								
*Study country*								
Asian	159	−0.35 (−0.67, −0.03)	0	0.9568	70	−0.34 (−0.82, 0.13)	NA	1.0000
Non-Asian	143	−1.11 (−2.10, −0.12)	85.5	0.0086	178	−1.59 (−4.00, 0.82)	97.4	<0.0001
								
*MetS diagnostic criteria*								
IDF	213	−0.85 (−1.66, −0.04)	87	0.0005	213	−1.66 (−3.84, 0.52)	97.6	<0.0001
Other	89	−0.36 (−0.80, 0.08)	NA	1.0000	35	−0.10 (−0.80, 0.60)	NA	1.0000
								
*Study treatment duration*								
2–3 months	204	−0.40 (−0.69, −0.12)	0	0.7932	150	−0.35 (−0.68, −0.02)	0	0.6478
6 months	98	−1.60 (−2.05, −1.14)	NA	1.0000	98	−4.14 (−4.85, −3.43)	NA	1.0000
								
*Jadad score*								
Low (<4)	143	−1.11 (−2.10, −0.12)	85.5	0.0086	178	−1.59 (−4.00, 0.82)	97.4	<0.0001
High(≥4)	159	−0.35 (−0.67, −0.03)	0	0.9568	70	−0.34 (−0.82, 0.13)	NA	1.0000

Abbreviations: BMI, body mass index; CI, confidence interval; IDF, International Diabetes Federation; MetS, metabolic syndrome; NA, not available.
